# Soft Materials with Time-Programmed Changes in Physical Properties through Lyotropic Phase Transitions Induced by pH-Changing Reactions

**DOI:** 10.1021/acsami.4c01455

**Published:** 2024-04-05

**Authors:** Emma Bowley, Wanli Liu, Dave J. Adams, Adam M. Squires

**Affiliations:** † School of Chemistry, University of Glasgow, Glasgow G12 8QQ, U.K.; ‡ Department of Chemistry, University of Bath, Bath BA2 7AY, U.K.

**Keywords:** stimuli-responsive materials, smart materials, pH-responsive materials, time-programmed materials, lyotropic liquid crystal, self-assembly

## Abstract

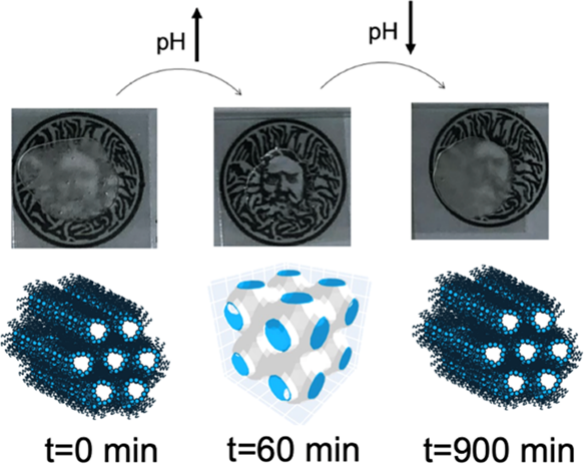

We present the development of time-programmable functional soft materials. The materials undergo reversible phase transitions between lyotropic phases with different topologies and symmetries, which in turn have very different physical properties: viscosity, diffusion, and optical transparency. Here, this behavior is achieved by combining pH-responsive lyotropic phases made from the lipid monoolein doped with 10% oleic acid, with chemical reactions that have well-defined controllable kinetics: autocatalytic urea–urease and methyl formate hydrolysis, which increase and decrease pH, respectively. In this case, we use small-angle X-ray scattering (SAXS) and optical imaging to show temporally controlled transitions between the cloudy hexagonal phase, which is a two-dimensional (2D) array of cylindrical inverse micelles, and the transparent, highly viscous three-dimensional (3D) bicontinuous cubic phases. By combining these into a single reaction mixture where the pH increases and then decreases again, we can induce a sequential transformation cycle from hexagonal to cubic and back to hexagonal over several hours. The sample therefore changes from cloudy to transparent and back again as a proof-of-concept demonstration for a wider range of soft materials with time-programmable changes in physical properties.

## Introduction

The ultimate goal of smart materials science is to have materials that can change their physical properties when we need them to. The main body of work to date has focused on these changes occurring in response to an external trigger. This work extends control to materials with properties undergoing programmable changes occurring with time.

Stimuli-responsive materials change their chemical/physical properties in response to changes in the surrounding environment.^1^ Suitable triggers include temperature,^2^ light,^3^ strain,^4^ and pH.^5^ Since stimuli-responsive materials adopt different behaviors depending on the conditions, desired properties can be programmed. As a result, stimuli-responsive materials hold considerable promise in applications ranging from disease detection^6^ to sustainable building environment development.^7^

Among the varying types of triggers, pH has attracted interest not only due to the ease of controllability but also the broad application potential.^8^ There are many examples of systems that respond to pH, including polymer systems with charged and ionizable groups that swell at specific pH^8,9^ and gels that form, change, or dissolve depending on the pH.^10−12^

However, these materials require the application of an external trigger. This makes them responsive rather than time-programmable. The addition of temporal control, as we describe here, opens up new applications: programmed changes in diffusion could allow the scheduled release of a drug or fragrance; and programmed switching in transparency, as in this work, has applications in packaging, indicating that a product has passed its expiry date. More fundamentally, temporal control underpins the design of complex systems. To quote Heinen and Walther:^13^ “if we plan to strive for active self-assembling systems with complex functionalities and life-like properties...we have to organize both in space and time”.

Amphiphilic molecules such as lipids self-assemble in water to form soft nanomaterials known as lyotropic liquid crystalline phases.^14^ Depending on conditions, a lipid mixture can adopt different geometry lyotropic phases,^15^ which differ dramatically in physical properties such as viscosity,^16^ optical transparency,^17^ and the diffusion and release of soluble molecules.^18^ These phases are thermodynamic equilibrium states, and the lipid systems respond to environmental changes, allowing reversible transitions between the different lyotropic structures.^19^

**Figure 1:**
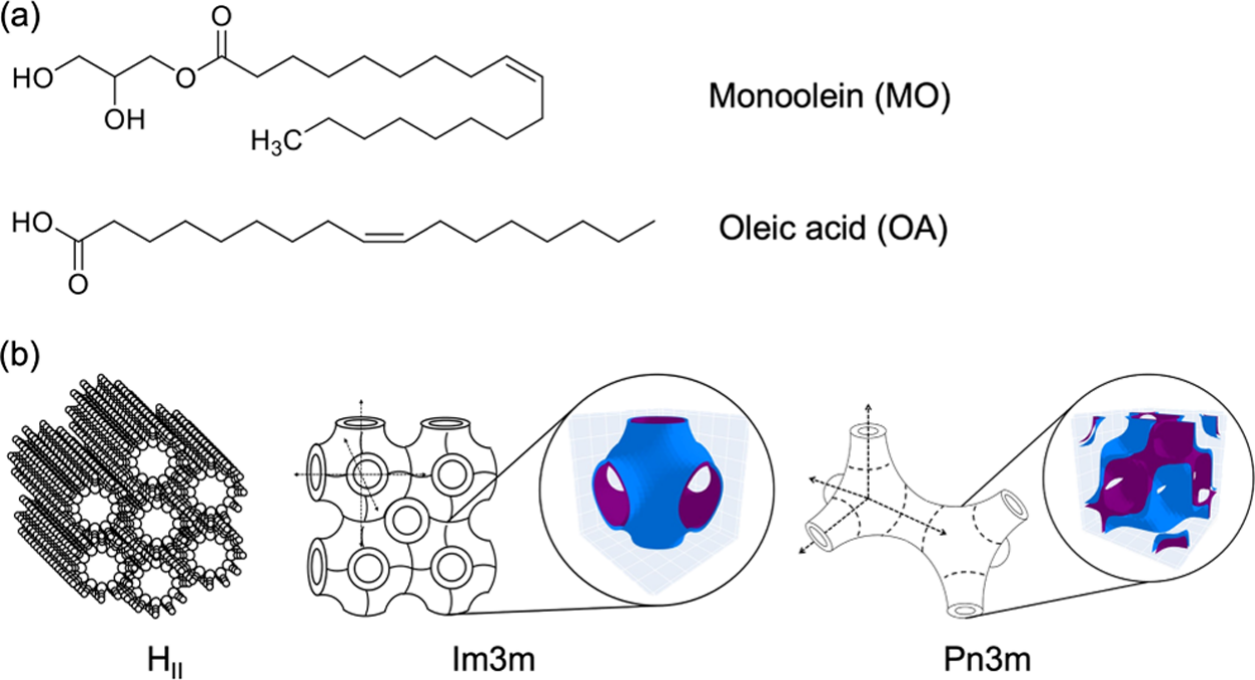
(a) Chemical structures of oleic acid and 1-monoolein; (b) three different phases shown by the 10%OA/MO system in excess aqueous at 25 °C: (left) inverse hexagonal phase (H_II_), (middle) primitive cubic phase (*Im*3*m*), and (right) double-diamond cubic phase (*Pn*3*m*). The insets highlight the lipid membrane (blue) and multijunction aqueous channels (purple).

1-Monoolein (MO) is one of the candidate lipid compounds that has been frequently used in reverse engineering and design of lyotropic crystal properties.^20^ MO is a ubiquitous lipid molecule. It is biocompatible, readily available, and widely used in pharmaceutical formulations and the food industry.^21^ MO comprises a polar glycerol headgroup that connects to oleic acid via an ester linkage (Figure 1). MO has been shown to form the following lyotropic phases depending on the temperature and the degree of hydration: planar monolayer/bilayer lamellar phases (Lα); disordered fluid of inverse micelles (L_2_); hexagonal (H_II_); micellar cubic (I_2_ or *Fd*3*m*); and bicontinuous inversed cubic phase structures including the primitive (*Im*3*m*), double-diamond (*Pn*3*m*), and gyroid (*Ia*3*d*).^15^

The present study presents temporally programmed transitions between the inverse bicontinuous cubic (*Pn*3*m* and *Im*3*m* symmetry)^22^ and inverse hexagonal (H_II_) phases.^23^ Both phases can exist under excess aqueous conditions, for example, as coatings^24^ or dispersed particles^25^ in water. The inverse lipid cubic phases are three-dimensional nanostructured materials formed by type II lipids^26^ arranged into a bilayer adopting a unique triply periodic minimal surface (TPMS), which has a constant mean curvature of zero and is periodic and continuous in the three spatial axes.^27,28^ The H_II_ phase consists of two-dimensional arrays of parallel cylindrical water channels, each surrounded by lipid monolayers. The cubic phases are optically transparent, nonbirefringent, and much higher in viscosity and diffusion than the opaque, birefringent H_II_ phase.^29^

The pH responsivity of lipidic materials can be engineered by the incorporation of a lipid with an ionizable headgroup, for example, a fatty acid. These systems are well-researched using lipid nanoparticles, and the feasibility of applying the systems as *in vivo* smart drug delivery cargoes has been demonstrated.^30−33^ pH-responsive lipid nanoparticles undergo morphological changes. For example, Xu et al.^34^ reported a MO-based nanoparticle system, which undergoes a phase change from an inversed hexagonal phase (H_II_) to a micellar cubic phase by increasing the surrounding pH from 4 to 7. The MO cubic phase needs to be doped with additional components to be susceptible to varying extents of the surrounding charges. Aota-Nakano et al. first reported a monoolein/oleic acid/pH buffer ternary system and studied the pH responsivity. Phase transitions of *Pn*3*m*–*Im*3*m* and H_II_–*Im*3*m* were both observed when increasing pH from 3 to 7, depending on the molar fractions of the deposited oleic acid.^35^ By employing the same principle, Negrini et al. developed another pH-responsive cubic phase system based on linoleic acid-doped monoolein.^36^

Research so far has mainly focused on the use of pH-induced lyotropic phase transitions such as the H_II_-cubic phase change for the triggered release of pharmaceutical compounds in biomedical applications, exploiting changes in diffusivity inside the aqueous channels. The wider range of physical properties that change, and application that these could give rise to, is relatively unexplored. For example, the fact that H_II_-cubic transition leads to an opaque-transparency appearance change^26^ provides implications for smart glass constructions.^37^

Furthermore, research so far has focused on responsiveness to an external trigger through a change in environment. Again, a wider range of applications can be realized by, instead, having phase transitions triggered by time. Indeed, there is significant interest in transient and dynamic changes in morphology and material properties in many gel systems, in many cases focusing on the potential life-like nature of such changes.^38−40^ For example, the group of Walther investigated a number of pH-driven systems.^41,42^ Lagzi et al. also reported a dynamic self-assembly system based on oleic acid and polymer building blocks using a pH switch.^43^ In this context, changes in lipid morphology in a tunable, dynamic fashion are also of interest. In this work, we will present preprogrammed temporal control over lipid self-assembly by coupling a pH-responsive lipid system with reaction-induced reversible pH changes.

## Methods and Materials

### Materials

Monoolein was purchased from Croda (Cithrol GMO HP-SO-LK, purity >96%). Sodium hydroxide (Honeywell), hydrochloric acid (Honeywell), urea (ultrapure 99%, Alfa Aesar), and urease (U4002–100 KU, Jack Beans, 100,000 units/g solid) were used as received. Methyl formate (anhydrous, 99%), sodium phosphate monobasic, and sodium phosphate dibasic were purchased from Sigma. All solutions were prepared using Milli-Q water (18.2 MΩ cm^–1^, Millipore, Bedford, MA). pH-adjusted 100 mM sodium phosphate buffer solutions were used throughout the experiment.

### Preparation of a 10%OA/MO (w/w) Matrix

MO was heated and sonicated at 60 °C in a water bath for 20 min to allow the transformation from solid to liquid/fluid-like.^15^ After this, 94 mg (approximately 100 μL) of MO was pipetted and subsequently mixed with 119 μL of ethanol to yield a mixture of 50/50 (w/w) monoolein/ethanol. This mixture was vortexed for 5 min to homogenize the sample. The procedure above was repeated for oleic acid (OA) to produce OA/EtOH, 50/50 (w/w). All samples were equilibrated at room temperature before use.

To prepare 10%OA/MO (w/w), 20 μL of 50%OA/EtOH was added to 200 μL of 50%MO/EtOH, followed by vortexing for 5 min. The lipid ethanol matrix was dried in a fume hood for 48 h to allow solvent evaporation. The Eppendorf tube that held the sample was weighed before and after evaporation to determine solvent loss.

### pH Measurement

pH measurements were performed using a HANNA FC200 pH probe with a 6 mm × 10 mm conical tip. Measurements were taken at room temperature. The accuracy of the pH values was ±0.1. To monitor the pH change of the system with urea, urease, and methyl formate, excess solution flowed through the SAXS system and was collected and measured for the duration of the SAXS measurements.

### Preparation of SAXS Measurement Samples

The 10%OA/MO sample was heated at 70 °C on a hot plate for 10 min, after which 40 μL of the sample was pipetted and injected into a capillary flow cell made in-house, where the X-ray beam passes through a 1 mm diameter quartz glass tube (wall thickness approximately 10 μm). Excess lipids were drained off, followed by gentle purging under N_2_ for 30 s to create a layer of coating. The mass of the lipid coating was obtained by weighing the capillary before and after. The thickness of the lipid layer could be estimated by assuming that it adopted a uniform cylindrical shape. The thickness was 61 ± nm (see the Supporting Information for calculation). The SAXS sample holder is not temperature-controlled, and the SAXS chamber temperature varies between 25 and 27 °C. Hence for all experiments, the temperature was in this range.

### Preparation Solutions for pH Triggering

Stock solutions of urea (2 M) and urease (0.253 mg/mL) were dissolved in H_2_O. For the urease stock solution, the concentration was calculated by taking the mass of the enzyme powder (in mg) dissolved in a known volume of H_2_O. HCl (0.1 M) and NaOH (0.1 M) were used to adjust the pH of the solutions. For the flow-through SAXS measurements, 3.2 mL of urease (0.254 mg/mL) was added to 0.8 mL of water, which had been adjusted to pH 4. This solution was then adjusted to < pH 4. Directly before the measurement, 20 μL of urea (2 M) was added to this solution, and the resulting solution was immediately used.

Methyl formate was stored in the fridge. For the flow-through SAXS measurements, 200 μL of methyl formate was added to 3.8 mL of water that had been adjusted to pH 10 to give a 5% (v/v) methyl formate solution, which was then immediately used.

For the combined trigger solution, 3.18 mL of urease (0.254 mg/mL) was added to 0.8 mL of water, which had been adjusted to pH 4. This solution was then adjusted to < pH 4. Directly before the measurement, 200 μL of methyl formate and 20 μL of urea (2 M) were added, and the resulting solution was used immediately.

### SAXS Instrumentation

Small-angle X-ray scattering data were collected with a sample–detector distance of 562 mm from an Anton Paar SAXSpoint 2.0 instrument using a Cu Kα radiation source (λ = 1.54 Å). Two-dimensional (2D) scattering patterns were acquired on a Dectris Eiger detector and reduced by azimuthal integration into one-dimensional (1D) radial profiles of intensity against the scattering vector using Anton Paar SAXSAnalysis software.

### In Situ Flow-Cell SAXS Measurement

The experimental setup is shown in Figure S1 in the Supporting Information. In short, 2 pieces of 1 m polytetrafluoroethylene (PTFE) tubing (Diba, 0.8 mm ID, 1.6 mm OD) were attached to both sides of a capillary that had been previously coated with OA/MO. The capillary was mounted onto the Anon Paar Multi-Capillary Heated-Cool Sampler. The tubing was fed out of the SAXS chamber; one end was placed into a 100 mL beaker containing a pH probe, while the other end was connected to a 10 mL syringe. The phase transformation induced by the pH reactions was monitored throughout three stages; before, during, and after the reactions took place.

Before the experiment began, 6 mL of buffer solution was dispensed into the capillary to hydrate the lipid coating. The pH of the buffer solution matched the starting pH of the hydrolysis reaction solutions. Five SAXS measurements with an exposure time of 60 s were collected consecutively to determine whether the system was equilibrated.

Prior to loading the reaction solution, the buffer solution inside the capillary was removed by injecting an empty 10 mL syringe. Approximately 4 mL of pH reaction solution was dispensed into the capillary shortly after it was emptied. The phase behavior of OA/MO soaked in the reaction solution was continuously monitored by using SAXS with an exposure time of 1 min for each frame over 20 min. After this, the exposure time was increased to 5 min per frame for the next 40 min. On the basis of previous data, we expected the rate of pH change to be at its peak for the first 20 min, after which the rate of pH changes gradually decreased.^10^ Therefore, a longer exposure time was used after the reaction took place for 20 min to obtain a better signal/noise ratio.

For the samples loaded with methyl formate solutions, the capillary was further monitored using SAXS overnight, with an exposure time of 20 min per frame because of the slow hydrolysis kinetics.^44^

### Polarized Light Microscopy and Optical Transparency Study

Polarized microscopy images were collected from a Motic PantheraTEC-BF. OA/MO cubic phase bulk paste samples were used for visualization under the microscope. Different pastes with varying compositions were prepared by mixing appropriate amounts of melted OA/MO with pH buffer solutions containing active pH switching components at a 60/40 (w/w) ratio. For each measurement, approximately 100 mg of paste was transferred onto a glass slide, followed by placing a coverslip on top of the sample. Pictures were taken at 0, 20, 60, and 15 h after the pH temporal control started.

For the optical transparency study, OA/MO pastes containing methyl formate and urea–urease solution were prepared at 60/40 (w/w, lipid/aqueous). A cloudy-clear-cloudy turbidity cycle over time was expected. After mixing up the sample, approximately 100 mg of the paste was transferred onto a cover slide at 0, 8, 20, 60, 180, and 15 h after the pH temporal control started. The thickness of the paste was controlled by covering the sample with an additional cover slide and gently squashing it to a thickness of 0.7–0.8 mm. Before taking photos, the samples were transferred onto three layers of watch glasses with a sheet that had the University of Bath logo underneath the bottom layer. The photos were taken from approximately 20 cm above the samples.

### Viscosity Measurements

Viscosity experiments were carried out on an Anton Paar Physica MCR 302 rheometer at room temperature. A CP25 cone and plate were used at a measuring distance of 0.047 mm. Viscosity sweeps were performed at a shear rate of 1 × 10 s^–1^. The 10%OA/MO sample was kept in a water bath at 25 °C in between measurements. For 1-point viscosity measurements, 100 μL of the 10%OA/MO sample was pipetted onto the plate, and 1 mL of the pH reaction solution was added to completely cover the 10%OA/MO sample. A viscosity sweep was run for 2 min at each time point, and the first data point was taken as the viscosity of the sample.

## Results and Discussion

### SAXS of 10%OA/MO in Varying pH Buffers

**Figure 2:**
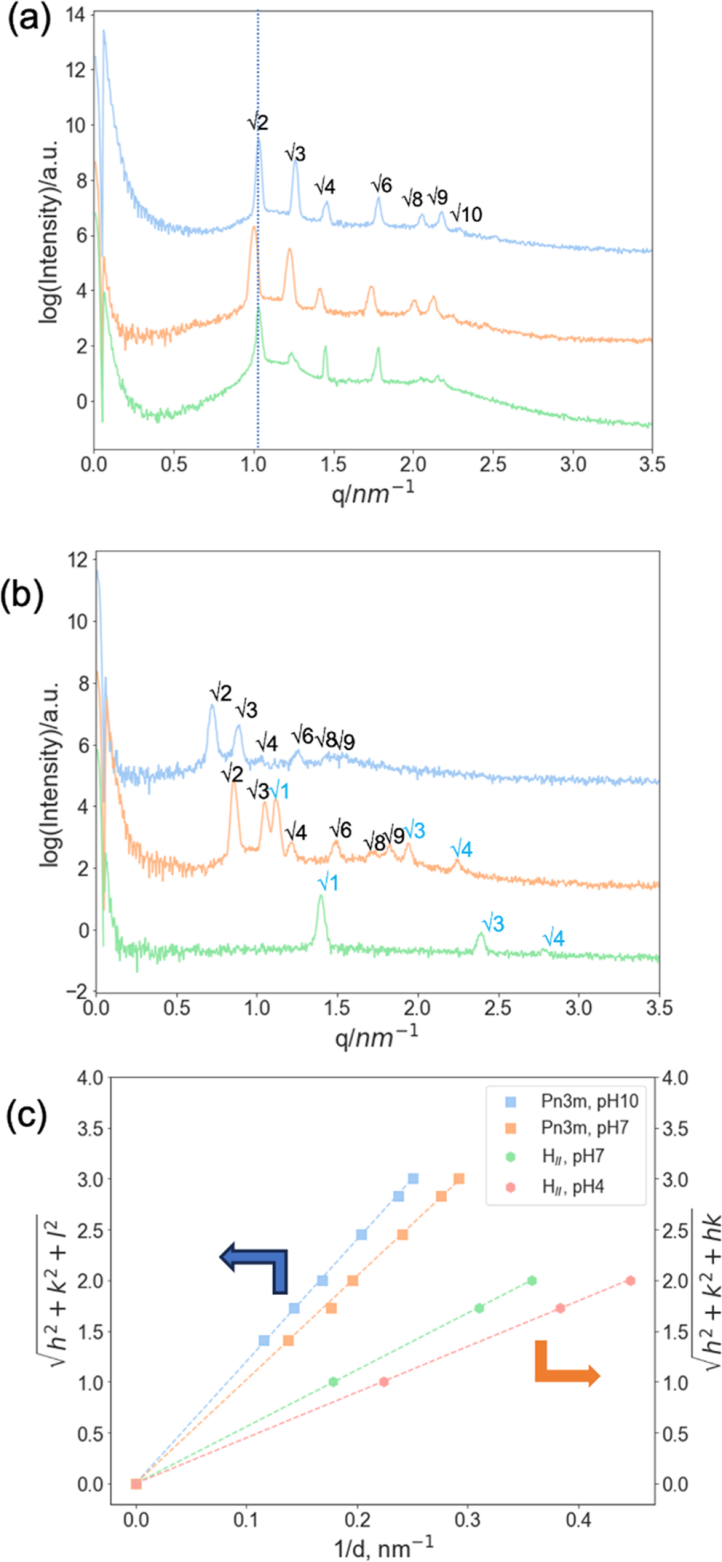
(a) SAXS patterns of MO in pH buffer solutions at pH 4 (green), 7 (orange), and 10 (blue) at 25 °C. The inserted dash line represents the 1st peak position of monoolein soaked in a pH 4 buffer solution; (b) SAXS patterns of 10%OA/MO soaked in three different pH buffers: 4, 7, and 10 at 25 °C (bottom to top), and (c) peak indexing for SAXS patterns in (b): the OA/MO cubic phases adopting *Pn*3*m* symmetry are indexed as √2, √3, √4, √6, √8, and √9; H_II_ phases are indexed as √1, √3, and √4.^26^

We first demonstrated that the phase behavior of the MO in an excess water environment was insensitive to any changes in pH. MO adopted *Pn*3*m* symmetries between pH 3 and pH 10 (Figure 2a). The peak positions of the mesophase were aligned at pH 3, 7, and 10, showing that the dimension of the mesophase was not sensitive to the change of the surrounding charges, consistent with data elsewhere,^35^ and to be expected due to the lack of charged or ionizable groups in the glycerol headgroup of monoolein (Figure 1).

To obtain pH-triggered changes, 10% oleic acid was added to the monoolein scaffold. 10%OA/MO (wt %) was placed in the same three buffers as the assays in the absence of OA. As can be seen in Figure 2b, varying phase behaviors were displayed. H_II_ and *Pn*3*m* (cubic) phases were observed at pH 4 and 10, respectively, while a mixed phase of H_II_ and *Pn*3*m* occurred at pH 7.

When the surrounding pH was lower than the p*K*_a_ of oleic acid (p*K*_a_ = 5),^45^ protonation of the oleic acid carboxyl headgroup occurs, removing the negative charge. The reduction in hydration and repulsion between headgroups on neutralization decreases the effective area of the hydrophilic headgroups of the oleic acid. This in turn makes the lipid/water interface more curved, producing the H_II_ phase observed at pH 4. Conversely, the headgroup area of lipids at the interface increases when the pH is higher than the p*K*_a_ of oleic acid, and the acid headgroup becomes negatively charged, producing a flatter interface of the cubic phases. Thus, 10%OA/MO adopts mixed *Pn*3*m*/H_II_ and *Pn*3*m* at neutral and alkaline pH, respectively. The peak positions for 10%OA/MO shift toward a smaller angle when the surrounding pH increases. This shift implies that the lattice cell dimension of the crystalline structure increases due to increased repulsion and decreased interfacial curvature (Figure 2c).

The pH-dependent phase behavior of other OA (wt %) compositions, i.e., 5, 15, and 20%OA, in pH 4, 7, and 10, was studied (see Figure S2). For the 15% and 20%OA lipidic matrices, the materials predominately adopt *H*_II_ in all of the pH conditions chosen, showing inertness toward pH. In contrast, 5% OA/MO showed pH-dependent phase behavior following a similar trend to 10%OA/MO. However, varying extents of the swelling effect caused by OA at pH 10 were observed (1st peak position of *q* ∼ 0.92 vs 0.72 nm^–1^ for 5% vs 10%OA/MO, wt %). Therefore, 10%OA/MO is chosen in this work to show a more dramatic pH-responsive phase behavior.

There are small differences in the phase behavior between our system and other work. Aota-Nakano et al. reported a phase transition of H_II_–H_II_ & *Pn*3*m*–*Im*3*m* for 10/90 OA/MO (mol %) by increasing the pH of the buffer solution from pH 3 to 7. The slight differences in the phase transition pH and adopted morphologies might be attributed to differences in impurities within the monoolein and oleic acid from different commercial sources.

### *Pn*3*m* to H_II_ Transition Triggered by Methyl Formate Hydrolysis

**Figure 3:**
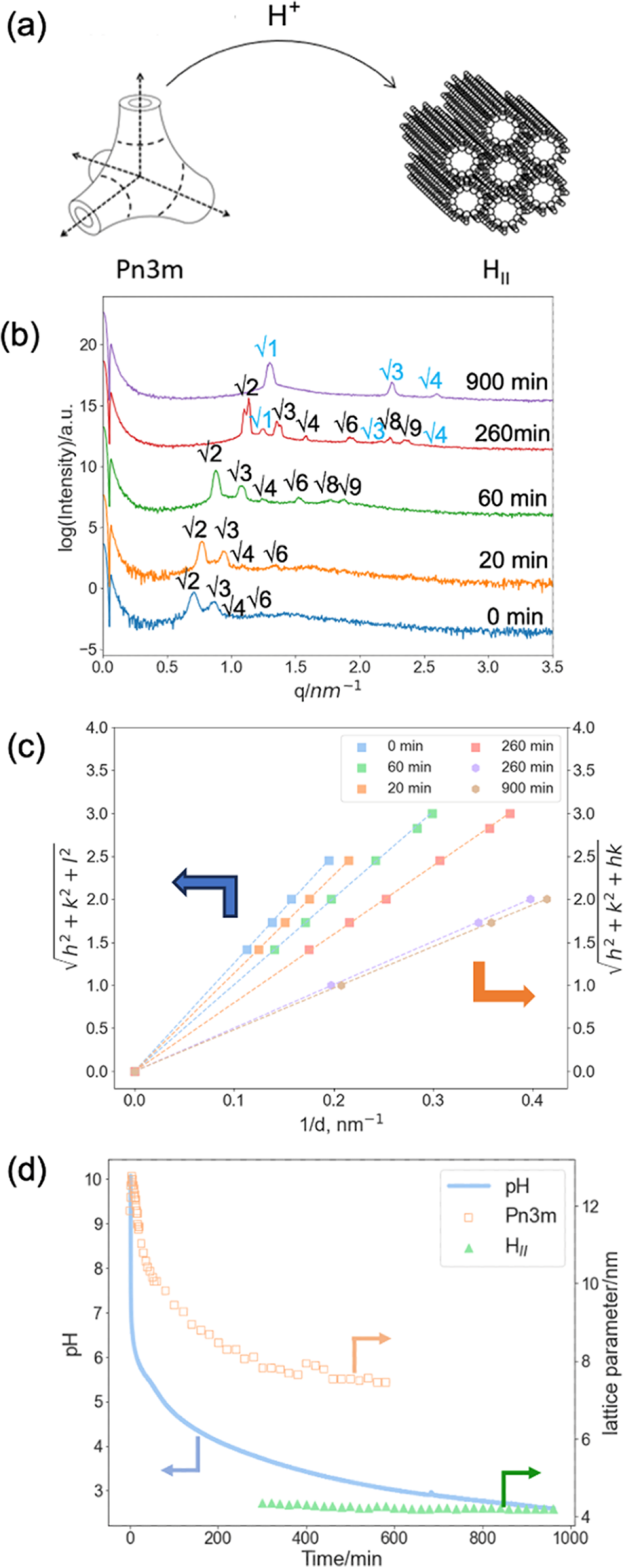
(a) Hydrolysis of methyl formate leads to a decrease in pH that triggers the phase transition of *Pn*3*m*–H_II_. (b) Time-resolved 1D SAXS patterns of 10%OA/MO in 5% methyl formate phosphate buffer (pH 10) to induce H_II_–*Pn*3*m* phase transition. (c) Peak indexing for SAXS patterns in (b): the OA/MO cubic phases adopting *Pn*3*m* symmetry are indexed as √2, √3, √4, √6, √8, and √9; H_II_ phases are indexed as √1, √3, and √4.^25^ (d) pH log of the reaction solution, the phase identity, and lattice parameter of the OA/MO mesophase during the methyl formate hydrolysis reaction.

The initial *Pn*3*m* phase was formed by soaking the OA/MO coating in a pH 10 buffer solution. To trigger the *Pn*3*m* to H_II_ phase transition (Figure 3a), we replaced the buffer solution with the 5% (v/v) methyl formate solution (pH 10). Hydrolysis of methyl formate at this pH results in a decrease in the pH to 4 over a period of approximately 16 h (hydrolysis of other esters and lactones would be expected to give similar results).^10^ The rate of pH change depends on the concentration of methyl formate.^10^ Here, we chose a 5% (v%) methyl formate phosphate buffer solution (pH 10) to facilitate a timed process. An in situ experiment was performed to monitor the phase behavior of OA/MO in the presence of a 5% methyl formate solution at pH 10.

As shown in Figure 3b (bottom), OA/MO adopted the *Pn*3*m* cubic phase for the first 20 min upon soaking in the methyl formate solution, and there were no signs of phase transition. The peak positions gradually shifted away from the origin, which suggested that the lattice cell dimensions were decreasing. A similar trend was also observed between 20 and 60 min after the reaction started, as displayed in Figure 3b (middle).

After leaving OA/MO in the methyl formate solution for 260 min, while the peak positions of *Pn*3*m* continued shifting away from the origin, a new peak was observed at *q* ∼ 1.3 nm^–1^. Note that the double peaks shown in the data at 260 min are real and may be due to a certain degree of alignment, as suggested by the 2D SAXS data (Figure S3). A set of new peaks was observed at *q* ∼ 1.2, 2.1, and 2.4 nm^–1^ from 300 min onward. The ratio 1:√3:2 is consistent with the H_II_ phase.^35^ The intensity of these new peaks increased, showing the increased formation of the H_II_ phase. The width decreased over time, either reflecting an increase in crystallite size according to the Scherrer equation^46,47^ or a decrease in sample heterogeneity; a range of slightly different lattice parameters across the sample would also cause peak broadening.

The pH values of the solution, phase identity, and lattice parameter of OA/MO are listed in Figure 3c. Both the pH value and lattice parameter decrease over time, as expected from the data in pure buffers (Figure 2). By the end of the reaction, the OA/MO had undergone a transformation from *Pn*3*m* to H_II_, with both phases coexisting at intermediate points. The H_II_ first appeared, and the *Pn*3*m* disappeared at pH 4 and pH 3, respectively.

The phase transition pH values are slightly different from those of the pH buffer assay, where the sample adopted the H_II_ phase alone at pH 4. This discrepancy may reflect out-of-equilibrium kinetic processes due to the hindered diffusion of formic acids or delayed time for the charge to be evenly distributed over the whole materials. Moreover, pH was not measured in the SAXS capillary itself but in the solution after flow-through, which may give discrepancies in the measured phase transition pH. This phenomenon was reproducible and will also be discussed in the following sections.

Overall, we successfully induced a slow transformation of OA/MO from *Pn*3*m* into H_II_ using a temporally controllable methyl formate hydrolysis reaction. The overall time scale of the phase transformation over many hours is of the same order of magnitude as the hydrolysis reaction driving it.

### H_II_ to *Im*3*m* Transition Triggered by Urea–Urease

**Figure 4:**
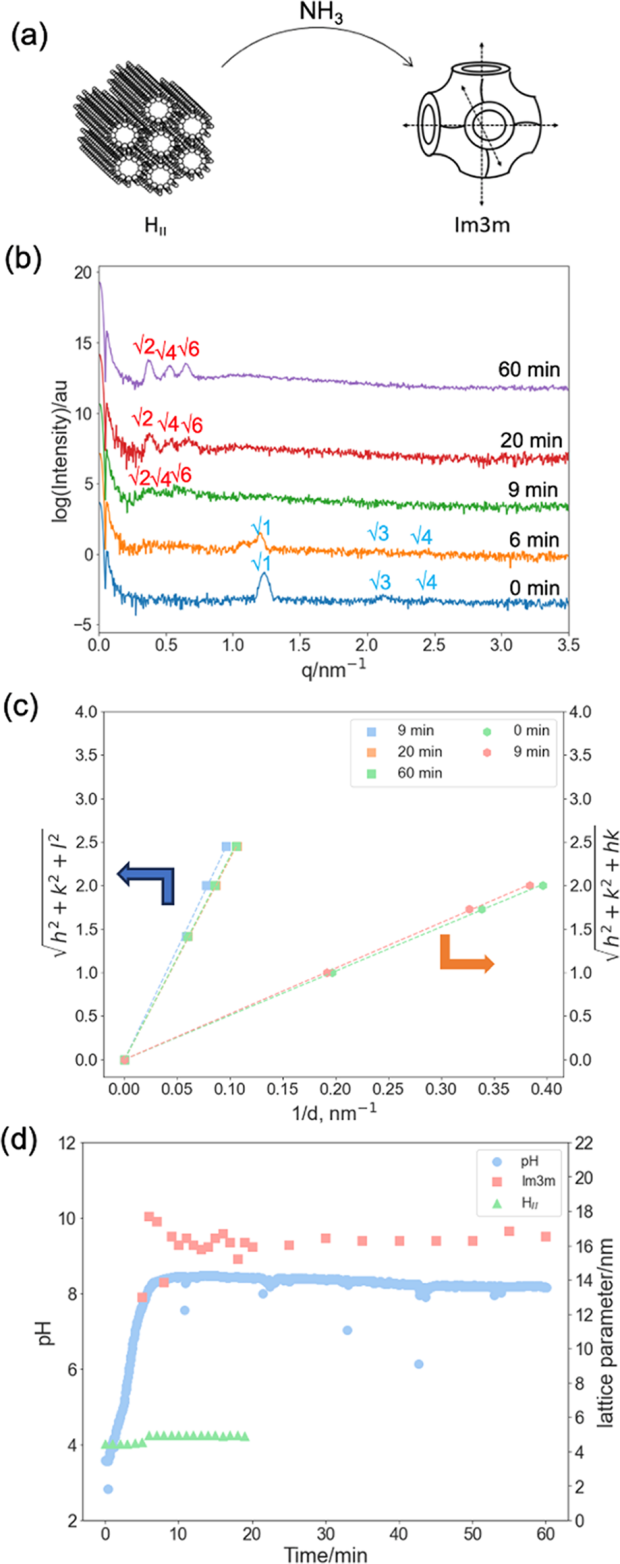
(a) Urea–urease autocatalytic reaction can be used to induce a phase transition of H_II_–*Im*3*m.* (b) Time-resolved 1D SAXS patterns of 10%OA/MO in urea–urease pH buffer solution (pH 4) to induce H_II_–*Im*3*m* phase transition. (c) Peak indexing for SAXS patterns in (b): the OA/MO cubic phases adopting *Im*3*m* symmetry are indexed as √2, √4, and √6; H_II_ phases are indexed as √1, √3, and √4. (d) pH log of the reaction solution, the phase identity, and lattice parameter of the OA/MO mesophase during the urea–urease autocatalytic reaction.

An initial H_II_ was formed by soaking OA/MO in a pH 4 buffer solution. The pH of a system can be increased using the urease-catalyzed conversion of urea to ammonia and carbon dioxide, resulting in a uniform increase of the pH to above 8.^10,48−50^ This can be used to trigger an H_II_–*Im*3*m* phase transition (Figure 4a,b). Starting at pH 4, OA/MO remains in the H_II_ phase for the first 20 min after the addition of the urea/urease solution. However, the peak intensity starts to decrease after 5 min until the H_II_ peaks disappear after 20 min. After 5 min, a new peak is observed at *q* ∼ 0.35 nm^–1^. Continuing from this, more peaks indicating the *Im*3*m* phase appear at *q* ∼ 0.55 nm^–1^ and *q* ∼ 0.7 nm^–1^ after 20 min. As seen with the methyl formate system described above, the new peaks increased in intensity over time as more *Im*3*m* was produced.

The pH values of the solution, phase identity, and lattice parameters of the OA/MO system as the system evolved are shown in Figure 4c. The lattice parameters do not change as the pH increases, rather a change in the phase is observed. As with the reaction with methyl formate described above, a mixed phase is seen during the overall transformation from H_II_ to *Im*3*m*, with the *Im*3*m* phase appearing at pH 8 and the H_II_ finally disappearing at pH 8.2. Again, there are a few differences from the equilibrium pH buffer assay behavior (Figure 2). First, the OA/MO system forms the *Im*3*m* cubic phase instead of the *Pn*3*m* cubic phase. The adoption of a different symmetry cubic phase may reflect a pathway-dependent trapped state; the *Im*3*m* and *Pn*3*m* phases are predicted to be similar in energy in excess water conditions,^51^ so there would be little driving force for their interconversion. Alternatively, the *Im*3*m* phase could be induced by an interaction with one of the species involved in the pH transformation. Second, the boundaries again occur at slightly different pH values; at pH 7, the transformation was still in the H_II_ phase, whereas it showed coexisting phases in the equilibrium pH 7 buffer earlier. Again, this may reflect the delayed kinetic processes or experimental aspects in the pH measurement discussed in the earlier experiment.

### H_II_ to *Im*3*m* to H_II_ Transition Triggered by Combining the Urease/Urea and Methyl Formate Reactions

**Figure 5:**
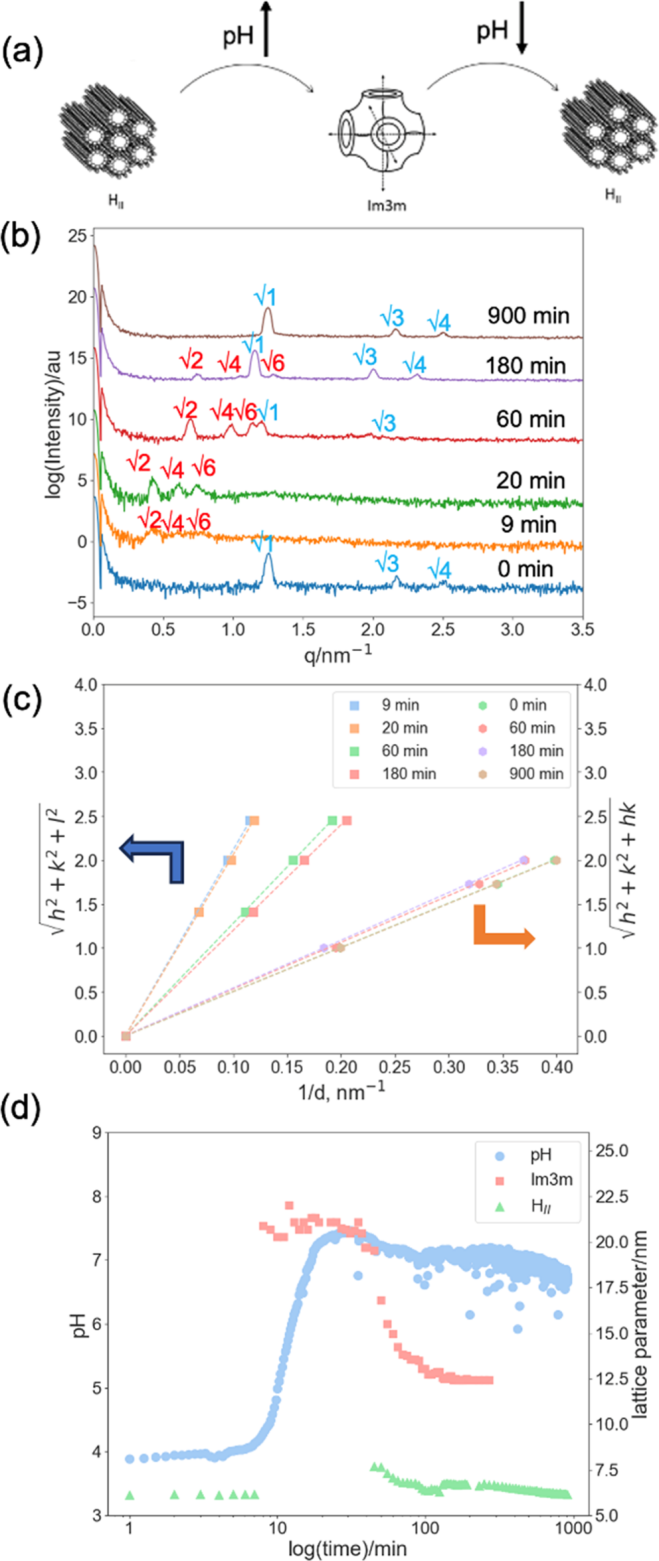
(a) Reaction scheme of the combined urea–methyl formate reaction. (b) Time-resolved 1D SAXS patterns for the OA/MO soaked in methyl formate, urea, and urease buffer solution with a starting pH of 4. (c) Peak indexing for SAXS patterns in (b): the OA/MO cubic phases adopting *Im*3*m* symmetry are indexed as √2, √4, and √6; H_II_ phases are indexed as √1, √3, and √4. (d) pH log of the reaction solution, phase identity, and lattice parameter of the OA/MO mesophase for the combined urea–urease and methyl formate reactions.

Finally, we combined both triggers to produce a single reaction mixture, inducing a phase transition from H_II_ to *Im*3*m* and back to H_II_ over a complete pH cycle. We started at pH 4. The urea/urease reaction is initially dominant as the reaction occurs at a much faster rate than the methyl formate hydrolysis. As such, the pH initially increases to around pH 8. As the urea is consumed, the methyl formate hydrolysis becomes dominant, and so the pH is reduced once again to pH 6.8 (Figure 5a). The time frame for this reaction was approximately 16 h. An in situ SAXS experiment was performed to monitor the phase behavior of OA/MO in the presence of both methyl formate and urea/urease solution.

The OA/MO system adopted the H_II_ phase for the first 10 min after the solution was injected (Figure 5b). The pH values of the reservoir, phase identity, and lattice parameter of OA/MO during the combined pH trigger experiment are shown in Figure 5c. As the pH increases, the *Im*3*m* peaks start to form at 9 min. This transition from H_II_ to *Im*3*m* appears not to proceed via coexisting phases, unlike the urea/urease system alone. This is likely due to the pH change being more rapid in this combined system, so we miss the transition. As expected, the cubic phase has the same Im3m symmetry as in the previous urea/urease experiment. After the next 2 h, the new cubic peaks increased in intensity and shifted to higher values of *q* over approximately 2 h (Figure 5b).

After 2 h, a second transition starts as the H_II_ peaks reappear. At this point, the *Im*3*m* peaks no longer shift, and the system remains in a mixed phase until the *Im*3*m* peaks disappear at 300 min. The peaks shifted to higher values of *q* as the system became more structured and the size of the crystalline material increased; this trend continued over 16 h. Both the pH and the lattice parameters decrease over time, as can be seen in Figure 5c, as expected from the methyl formate reaction described above. Hence, we are able to combine both reactions to access a two-step transition of *Im*3*m–Im*3*m* and H_II_–H_II_. The phase transitions of OA/MO shown are dependent on the rate of methyl formate hydrolysis and the urea/urease reaction, which triggered the pH of the solution to increase and decrease. It is worth noting that it has been shown for work on gel-based systems that the rate of pH change can be tuned (both up and down independently) by variation of any of the concentrations of urea, urease, and methyl formate.^10^

### Optical Transparency and Viscosity Study

**Figure 6:**
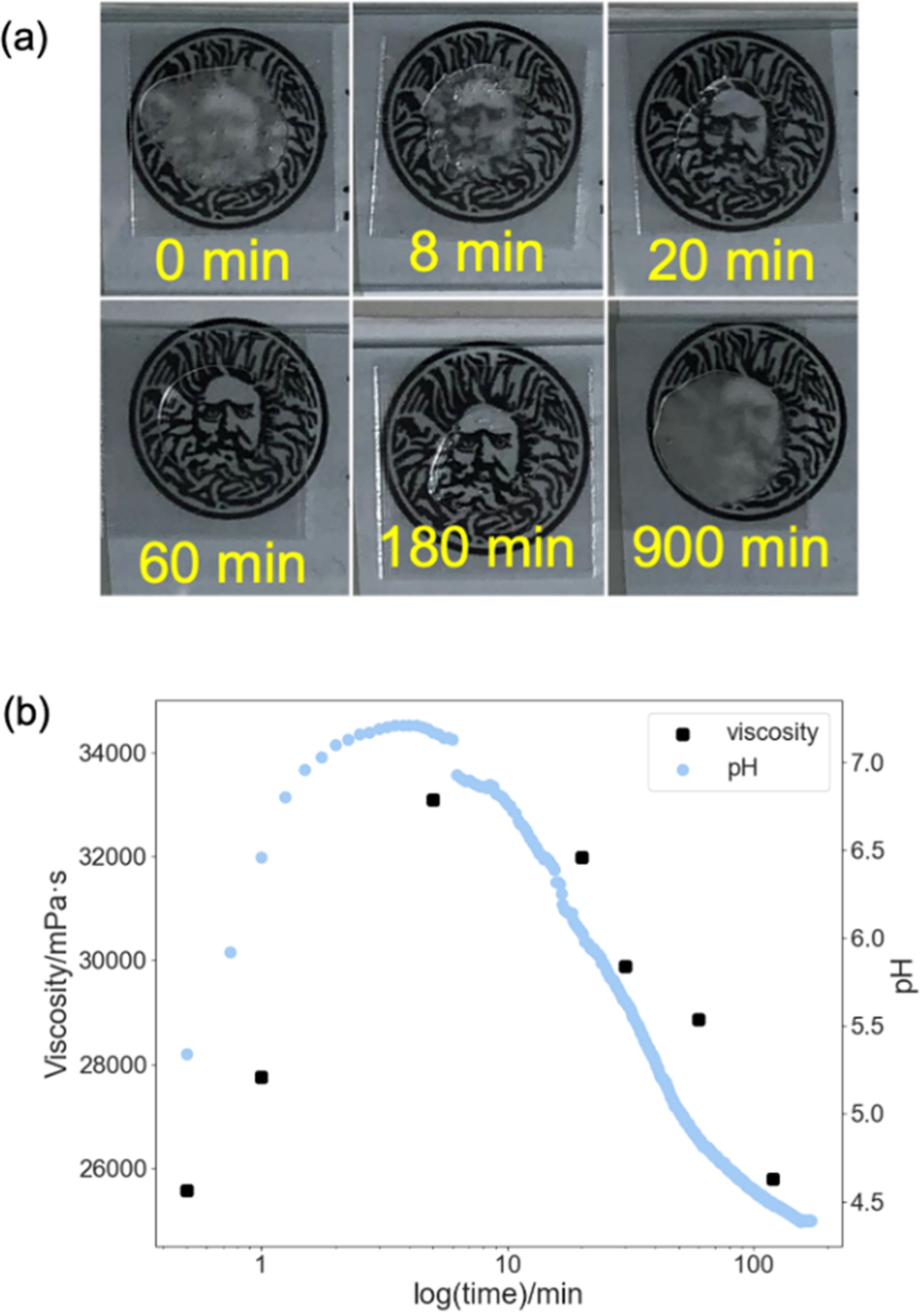
(a) Changes in turbidity over time using the combined urea–methyl formate reaction. The cover slides are 20 mm × 20 mm, and the diameter of the logo is 18 mm. (b) Changes in viscosity with time along with the changes in the pH of the system used.

The phase transitions described above are useful as the H_II_ and cubic phases can be easily distinguished because of the differences in birefringence.^26^ Images were taken following the same time point as the SAXS experiments described above. The results for the combined urea–urease and methyl formate triggered pH switching assay are displayed in Figure 6a. The OA/MO looked opaque initially at pH 4 (Figure 6a). The microscopy image showed birefringence under a polarizing filter, implying that the OA/MO adopted the H_II_ phase (Figure S8). As the urea–urease autocatalytic reaction proceeds, after 20 min, the sample becomes transparent (Figure 6a) and the magnitude of birefringence is lowered (Figure S8). These changes are due to the system becoming more isotropic as the cubic phase predominated. The sample becomes completely transparent and dark under cross-polarizers after 60 min. Using polarized light to determine the phase state of monoolein is a well-established method and has been used for decades to identify lipid mesophases.^31^

As the pH decreased once again, the sample regained turbidity, and the birefringence increased once again (Figures 6a and S8). Similarly, the H_II_ phase and cubic phase are expected to have different viscosities. This is indeed what we find; removing samples from the system over time shows that initially the sample is relatively nonviscous. As the pH increases, the viscosity increases as the cubic phase is formed. Then, as the pH decreases once again, the viscosity drops back once again to close to the original value. These changes in turbidity and viscosity agree with the expected results from flow-through SAXS experiments. Hence, we can use this preprogrammed temporal control over lipid self-assembly to prepare useful materials with controlled macroscopic properties.

## Conclusions

We have successfully shown a new pathway for the following transitions: *Pn*3*m* to H_II_, H_II_ to *Im*3*m*, and H_II_ to *Im*3*m* and back to H_II_ in the 10% oleic acid monoolein system. The system is pH-responsive and, therefore, capable of self-assembling into different crystalline phases when the pH changes. Therefore, the autocatalytic urea–urease reaction and the hydrolysis of the methyl formate reaction were used to achieve these transitions. The process presented here shows promise for changing the systems’ macroscopic properties over a desired period and has many possible applications for material development.

## Supporting Information Available

Full description of the flow-through SAXS experimental setup; polarizing microscopy images for methyl formate triggered pH switching assay; urea–urease triggered pH switching assay; and methyl/urea–urease combined pH switching assay (PDF)

## Abbreviations

MO: monoolein
OA: oleic acid
