# Supplementary material for: Soft Materials with Time-Programmed Changes in Physical Properties through Lyotropic Phase Transitions Induced by pH-Changing Reactions
Source: ACS Appl Mater Interfaces. 2024 Apr 5;16(15):19585–93. doi: 10.1021/acsami.4c01455 (PMC11040581; doi:10.1021/acsami.4c01455)
Supplement: Supplementary file 1 — am4c01455_si_001.pdf [file am4c01455_si_001.pdf]

SUPPORTING INFORMATION

Soft materials with time-programmed changes in physical properties through lyotropic phase transitions induced by pH-changing reactions

Emma Bowley, [‡a] Wanli Liu, [‡b] Dave J. Adams[*a] and Adam M. Squires[*b]

a. School of Chemistry, University of Glasgow, Glasgow, G12 8QQ, U.K.
b. Department of Chemistry, University of Bath, Bath, BA2 7AY, U.K.

*Corresponding authors:

Dave.Adams@glasgow.ac.uk (Dave Adams); A.Squires@bath.ac.uk (Adam Squires)

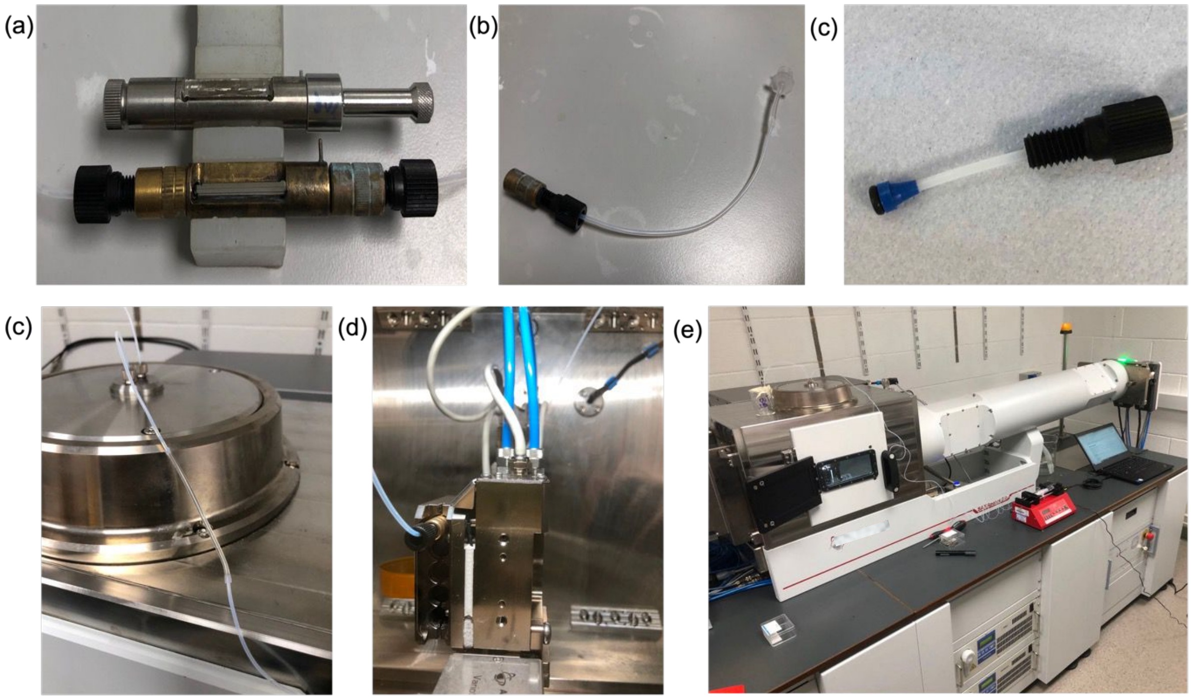

Figure S1. Set up for In-situ flow-cell SAXS. (a) Top: a quartz capillary from Anton Paar with metal disk seals, bottom: a 1:1 house-made metal case holds a borosilicate capillary (ø=0.7mm, thickness=0.1mm) with open-ended disk seals, (b) an example of the metal disk seal connects to a PTFE tubing for flow-through experiments, (c) an example of the flow-through tubing connection; an ETFE ferrule (1/16" OD) and the corresponding end fitting were threaded through the PTFE tubing, and an O-ring (ID=1 mm) was also attached to the end faces the capillary, (d) the PTFE tubing were fed into the SAXS chamber, and an Anton Paar heat-cool sampler holds a flow-through capillary, and (e) overall looking of the set-up.

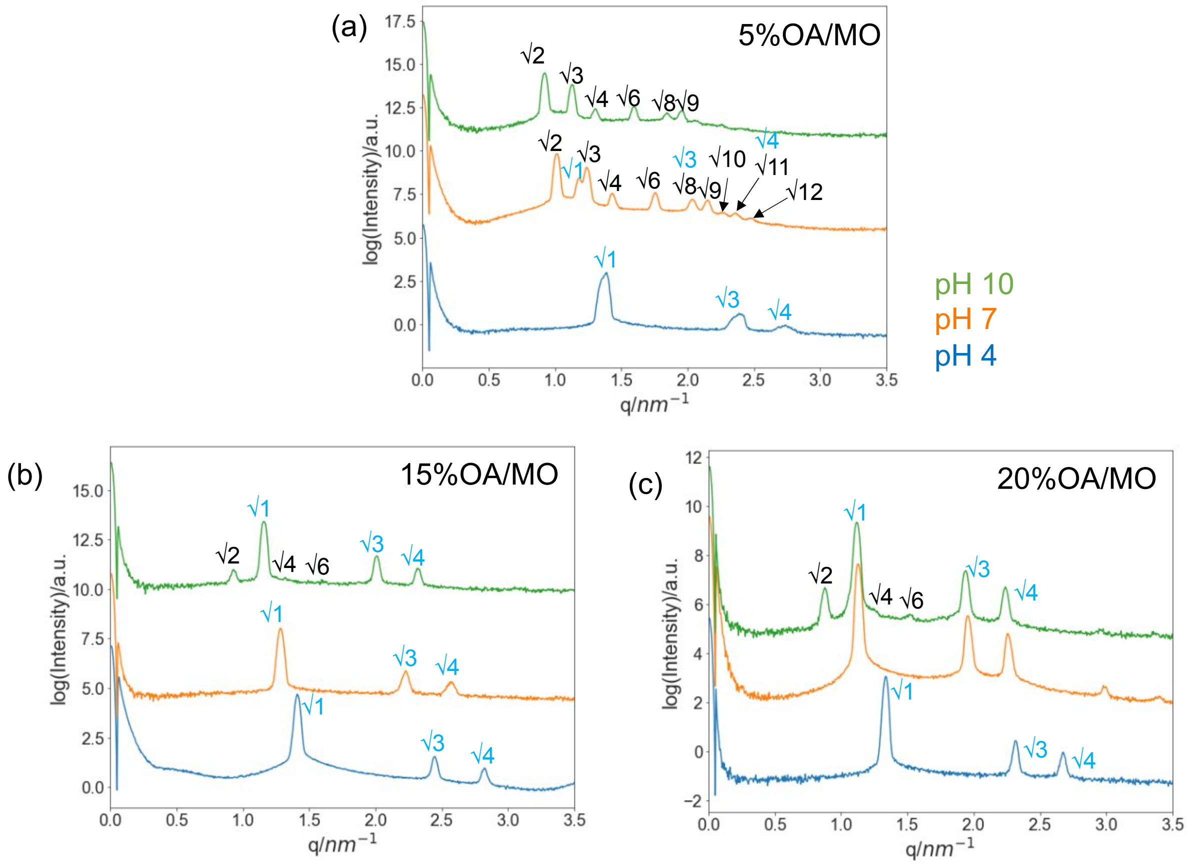

Figure S2. 1D SAXS patterns for (a) 5%OA/MO, (b) 15%OA/MO and (c) 20%OA/MO in pH4 (blue), pH7 (orange) and pH10 (green)

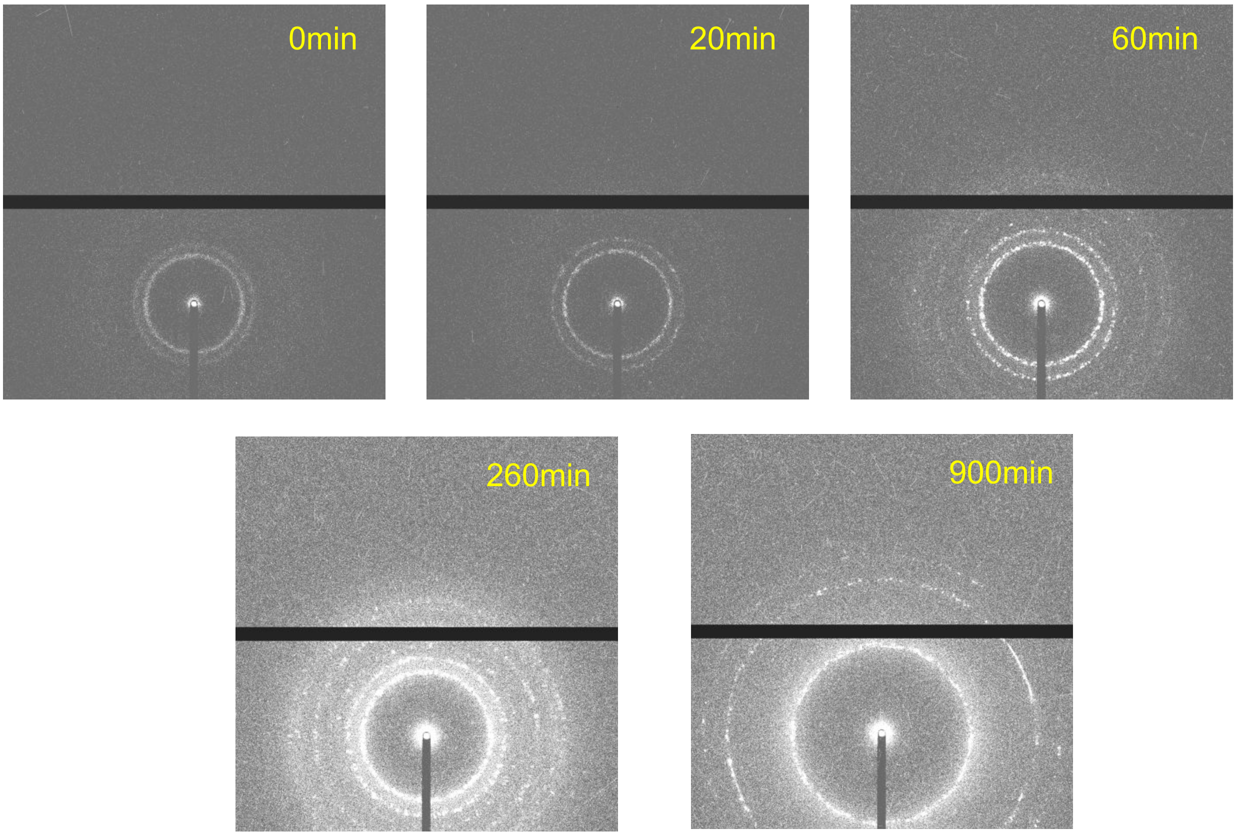

Figure S3. 2D SAXS patterns of the OA/MO coating soaked in the methyl formate solution at varying time point after the pH switching reaction started. The processed 1D patterns using azimuthal integration are shown in Figure 3b in the article.

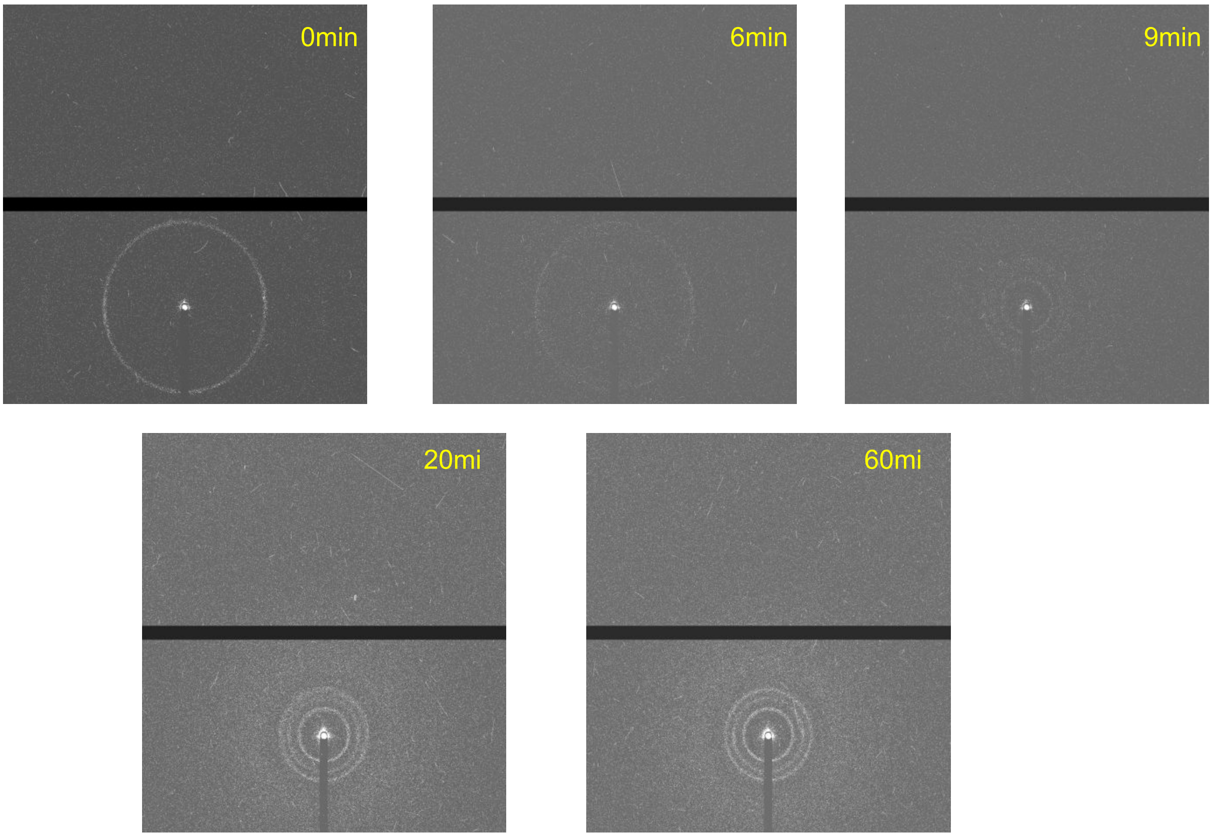

Figure S4. 2D SAXS patterns of the OA/MO coating soaked in the urea-urease solution at varying time point after the pH switching reaction started. The processed 1D patterns using azimuthal integration are shown in Figure 4b in the article.

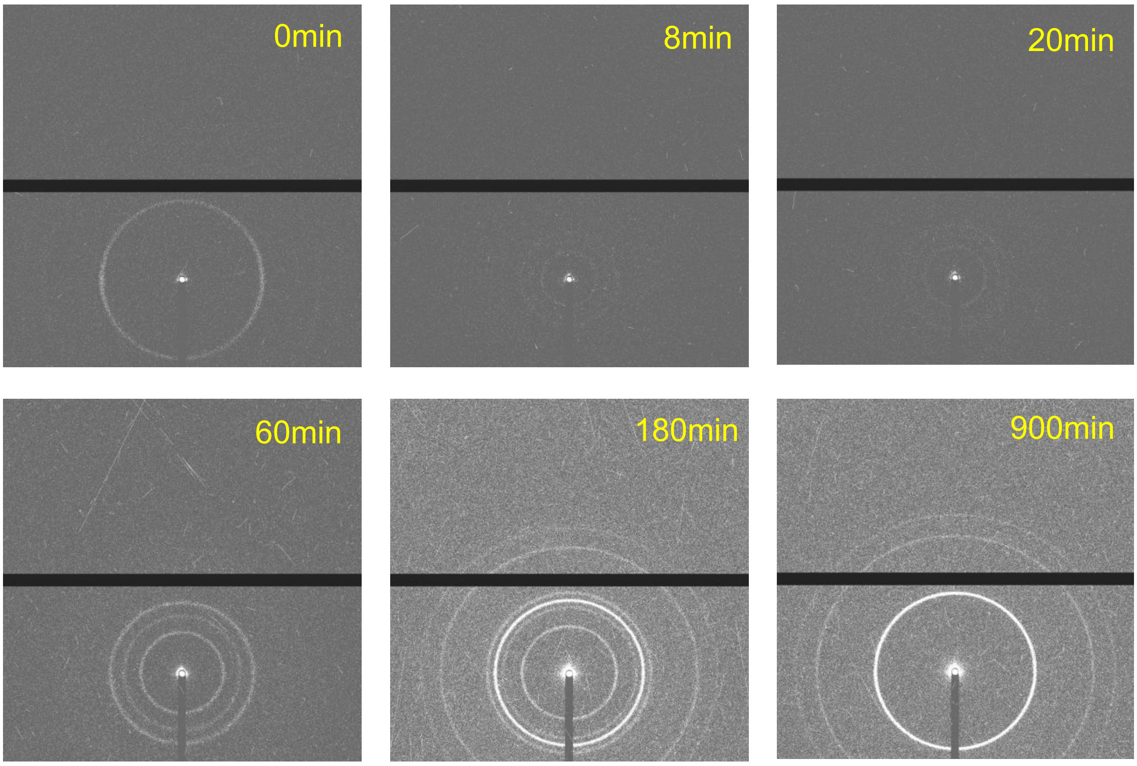

Figure S5. 2D SAXS patterns of the OA/MO coating soaked in the urea-urease/methyl formate solution at varying time point after the pH switching reaction started. The processed 1D patterns using azimuthal integration are shown in Figure 5b in the article.

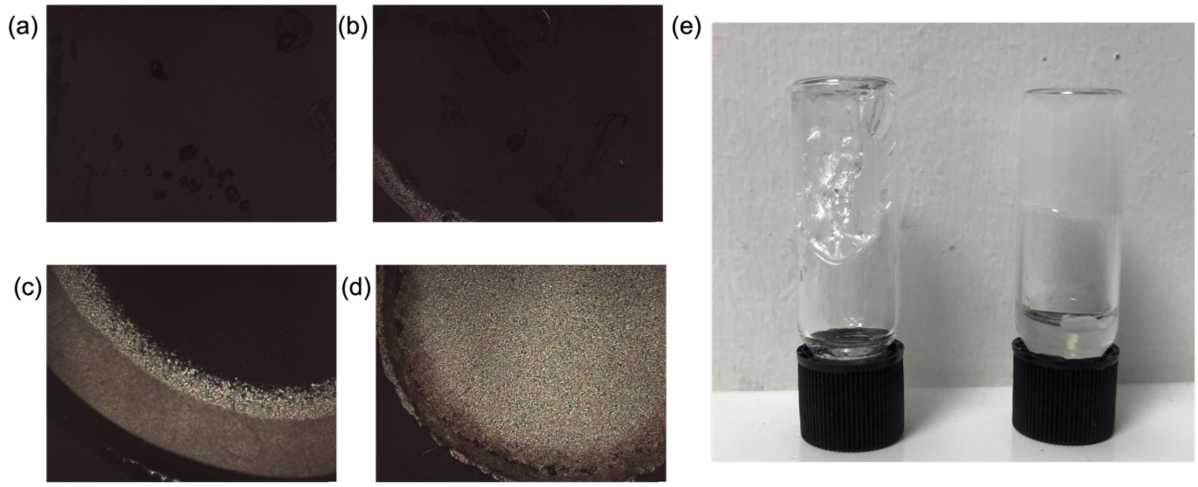

Figure S6. Polarized-light microscopy study on the 10%OA/MO cubic phase bulk paste in excess methyl formate pH10 solution, in which the same reaction condition as the through-flow SAXS experiment was applied. Microscopy images were taken at (a) 0 min, (b) 60 min, (c) 180 min and (d) 900 min after the cubic phase sample had been prepared, and (e) the macroscopic appearance of the matrix at 0 min (left) and 900 min (right) after the reaction started, a change in turbidity of the system can be observed.

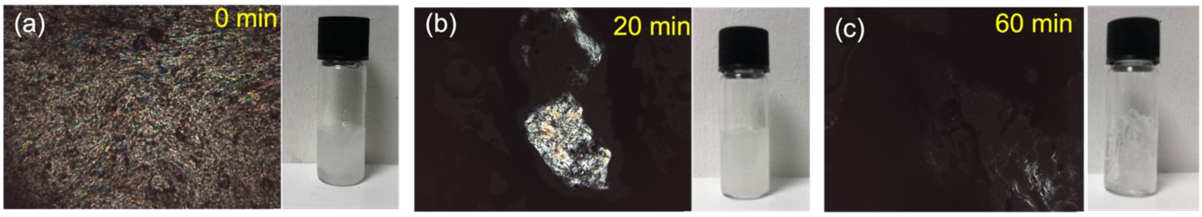

Figure S7. Polarized-light microscopy study on the 10%OA/MO cubic phase bulk paste in excess urea-urease pH4 solution, in which the same reaction condition as the through-flow SAXS experiment was applied. Microscopy images and pictures was taken at (a) 0 min, (b) 20 min, (c) 60 min after the reaction started, a change in turbidity of the system can be observed.

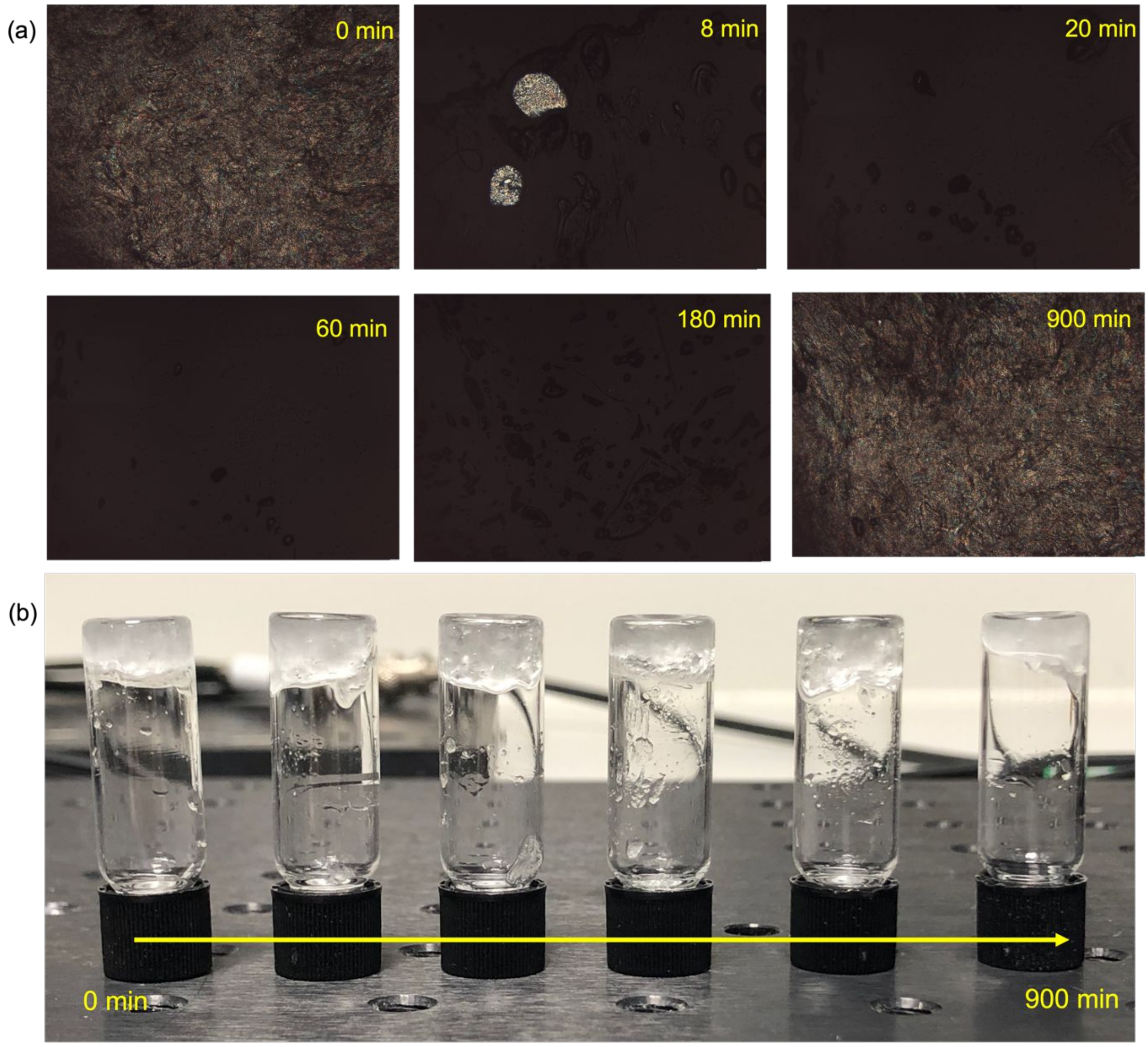

Figure S8. (a) Microscopy images under polarized light of the OA/MO matrix prepared in urea-urease/methyl formate solution at varying time point after the pH switching reaction started, and (b) photographs showing the macroscopic appearances of OA/MO soaked in the urea-urease/methyl solution over the course of the closed-loop pH switching reaction.
